# Assessment of percutaneous closure for decannulation of veno-arterial extracorporeal membrane oxygenation: A retrospective study

**DOI:** 10.1177/11297298241300119

**Published:** 2024-11-24

**Authors:** Diana Martins-Fernandes, João Rocha-Neves, Ana Rita Ferreira, Hélio Martins, Sérgio Gaião, José Artur Paiva

**Affiliations:** 1 Department of Emergency and Intensive Care Medicine, São João University Hospital Centre, Porto, Portugal; 2 Department of Biomedicine – Unity of Anatomy, Faculty of Medicine, University of Porto, Porto, Portugal; 3 RISE@Health, Rua Dr. Plácido da Costa, s/n, Porto, Portugal; 4 Faculty of Medicine, University of Porto, Porto, Portugal

**Keywords:** Mechanical circulatory support, decannulation, suture-mediated vascular closure devices, percutaneous closure, Perclose Proglide^®^, procedure-related complications

## Abstract

**Background:**

Despite the evidence supporting the use of Perclose Proglide^®^ (PP) suture-mediated vascular closure devices in various clinical scenarios, limited evidence exists regarding its role in percutaneous arterial closure of the femoral artery after venoarterial extracorporeal membrane oxygenation (VA-ECMO). Compared to conventional methods, this study evaluates the effectiveness and complications of bedside percutaneous femoral artery closure using Perclose ProGlide (PP) for VA-ECMO decannulation.

**Methods:**

Retrospective cohort of consecutive patients managed with mechanical circulatory support VA-ECMO for refractory cardiogenic shock, who survived decannulation between January 2017 and August 2023. A comparison between PP and other decannulation strategies was established to evaluate the effectiveness and procedure-related complications of bedside percutaneous femoral artery closure using a PP with a post-closure technique compared to conventional approaches of surgical and manual decannulation.

**Results:**

Among 122 patients decannulated from VA-ECMO with a mean age of 48.6 ± 13.1 and 78 (63.9) males, 49.2% comprised the PP group. Despite the older age (*p* = 0.021) and higher prevalence of arterial hypertension (*p* = 0.045), the PP group had a larger number of patients free from decannulation-related adverse events. Additionally, a higher haemoglobin level 24 h post decannulation (*p* = 0.047), with no difference in terms of transfusion of red blood cells between groups (*p* = 0.263) was found. The pseudoaneurysm was the most frequently reported complication, while the arterial cannulation surgical wound site infection was only documented in the open repair subgroup. A trend towards reduced Intensive Care (ICU) and hospital length of stay after decannulation was noted, although it did not reach statistical significance. There was no difference in mortality between both groups and no procedure-related deaths occurred. A mean of 2.7 PP devices were required to achieve complete haemostasis in the PP cohort, where technical failure was documented in four cases (6.7%).

**Conclusions:**

Bedside Percutaneous decannulation of VA-ECMO using a PP device with a post-closure technique is safe and reliable for achieving effective haemostasis, with fewer vascular complications than conventional approaches and a low device failure rate.

## Background

The use of extracorporeal membrane oxygenation (ECMO) has increased exponentially over the past decade, with a substantial rise in both Extracorporeal Life Support Organization (ELSO) centres and ECMO-runs by ELSO centres per year.^2^ The increase has resulted from expanded indications, improved management strategies and patient selection, enhanced cannulation techniques and device technology, all within streamlined processes and dedicated ECMO teams, which increase experience and improve overall outcomes.^3^

Peripheral VA-ECMO cannulation via the common femoral artery and vein, with the distal perfusion catheter placed ipsilateral to the cannulated femoral artery, is the most common approach.^1,2^ Despite improved cannulation techniques, arterial cannulas carry the risk of vessel injury, and vascular complications during decannulation are not uncommon.^2^ Among adult patients, the rates of limb ischaemia, fasciotomy and amputation are high, ranging from 0.156 to 0.410 per 1000 ECMO hours.^1^ Although these are well documented, more guidance is needed for decannulation procedures. The latest ELSO Interim Guidelines for VA-ECMO recommend surgical decannulation for large-sized cannulas (19–21 Fr),^2^ which are rarely used. Moreover, surgical decannulation is not without pitfalls, including impaired wound healing, surgical site infection, delayed mobility and increased costs associated with operating theatre times.^3,4^

The imperative of achieving rapid haemostasis, minimizing arterial complications and reducing the process complexity remain the rationale for the introduction of vascular closure devices. Despite the array of available devices, the safety and effectiveness of vascular closure devices for decannulation of VA-ECMO remain controversial and is yet to be definitively proven. Among these, the Perclose Proglide^®^ system (PP – Abbott Vascular, CA, USA) suture mediated device, endorsed for closure of arteriotomy sites ranging from 5 to 21 Fr, are designed to suture arteriotomy sites via a percutaneous approach. It works by insertion over a guidewire until blood flow confirms correct positioning, subsequently deploying ‘feet’ to anchor against the vessel wall, followed by needle deployment to create a suture loop, effectively closing the arteriotomy upon tightening.^5^ The PP has proven to be efficacious and cost-effective following cardiac catheterization,^6^ and holds a high technical success rate with no device or procedure-related major adverse events in the setting of endovascular aortic repair.^7^ Despite the evidence supporting its use in other clinical scenarios, its role in arterial percutaneous closure in VA-ECMO decannulation has been described, but data directly comparing PP percutaneous closure with conventional strategies is scarce.

Considering the absence of a standard procedure for VA-ECMO decannulation, the limited experience and evidence for percutaneous arterial closure of the femoral artery the authors conducted a retrospective evaluation of the effectiveness and procedure related complications of bedside percutaneous femoral artery closure using PP with a post-closure technique for VA-ECMO decannulation, in comparison with conventional strategies.

## Methods

**Figure 1.**
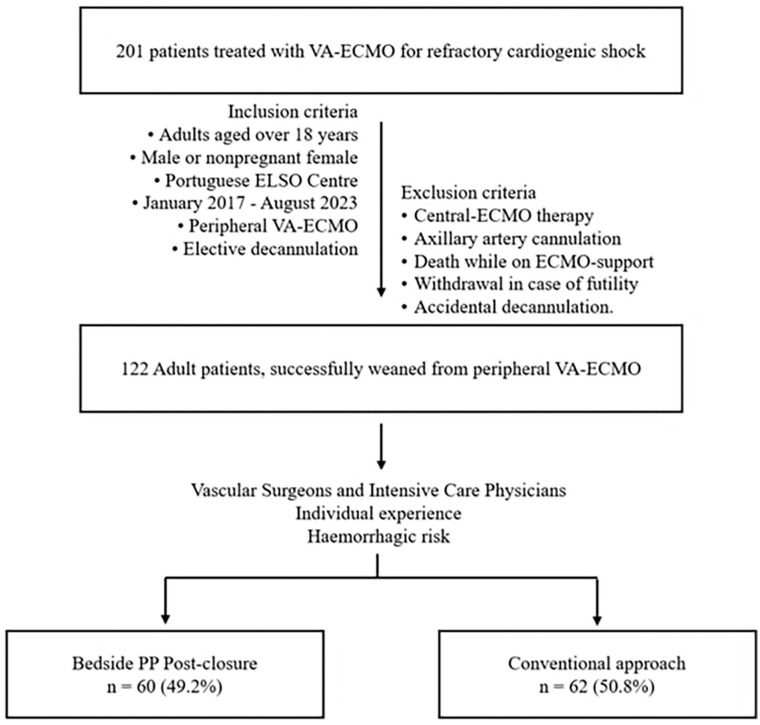
Flow chart of the study. VA-ECMO: venoarterial extracorporeal membrane oxygenation.

A retrospective cohort of consecutive patients managed with mechanical circulatory support via VA-ECMO for refractory cardiogenic shock, who survived to decannulation, between January 2017 and August 2023, at a tertiary referral centre, was selected. A flow chart of the study is provided in Figure 1. Inclusion criteria encompassed adults aged over 18 years, male or nonpregnant female, who were successfully weaned from peripheral VA-ECMO therapy. Exclusion criteria included central-ECMO therapy, axillary artery cannulation, death while on ECMO-support, withdrawal in case of futility and accidental decannulation. Considering the technique employed for femoral artery closure, patients were divided into a post-closure PP group and an historical conventional group (control group), encompassing manual compression and surgical repair. Decannulation of the venous and distal reperfusion cannulas were not the study’s focus.

The primary outcome was decannulation-related adverse events. Secondary outcomes included technical failure, post-decannulation ICU and inward length of stay, red blood cell transfusion and mortality within 30 days after ICU discharge.

Adverse events related to the decannulation procedure were defined as major or persistent bleeding (persistent oozing after initial manual compression, haemodynamic compromise, transfusion of two or more units of red blood cells or a fall in haemoglobin of more than 3 g/dL), haematoma requiring intervention, limb ischaemia, pseudoaneurysm, fistula, arterial thrombosis and access site infection. Procedural success was defined as complete haemostasis after PP deployment without manual compression beyond 5 min, with no additional surgical or endovascular repair. Device failure was defined as the inability of the device to achieve haemostasis due to suture breakage during plunger withdrawal, or the need for surgical revision of the femoral artery in post-decannulation period.

This study is under the framework of the Strengthening the Reporting of Observational Studies in Epidemiology (STROBE) 2014 Guideline.^8^ Data Collection, including patients’ demographic, preprocedural, procedural and outcome related variables were obtained from a review of the patient’s electronic health records and processed anonymously. The institutional Ethics Committee reviewed and approved the study and waived the requirement for patient consent (Approval number: CES 189/2024). All the procedures followed the ethical standards and complied with the Helsinki Declaration. Data processing was conducted anonymously, adhering to the principles outlined in the European General Data Protection Regulation.

### Setting and population

The centre operates an intensivist-led ECMO programme since 2010, with a dedicated ICU team, consisting of intensive care medicine specialists, with immediate availability of an ECMO-trained intensivist 24 h per day. They are responsible for the decision to introduce ECMO support, manage the patient and ECMO-related complications, and decide about weaning and liberation from ECMO support, including performing ECMO decannulation.

VA-ECMO was considered in refractory cardiogenic shock, in patients with reversible cardiocirculatory collapse or eligible to alternative cardiocirculatory assistance (ventricular assist devices or transplantation). Cardiogenic shock, suitable for VA-ECMO was generally characterized by systolic blood pressure less than 90 mmHg with clinical and laboratory evidence of end-organ damage, unresponsive to optimal conventional treatment. Relative contraindications included unrecoverable cardiac function not candidate for alternative cardiocirculatory assistance, poor life expectancy (end-stage organ disease, malignancy, etc.), severe neurologic impairment (extensive acute brain injury or prolonged anoxic brain damage), severe vascular disease and severe immunologic disease with marked blood and coagulation disorders. Age alone was not a contraindication. Aetiologies affecting VA-ECMO function (e.g. aortic regurgitation) were considered in an individual basis.

Percutaneous cannulation was performed by a trained intensivist using a modified Seldinger technique with a standard arterial cannula size ranging from 13 to 17 Fr, placed via the common femoral artery. Arterial and venous cannulas were, preferably, placed in separate limbs. To minimize distal limb ischaemia, a 6–8 Fr distal reperfusion cannula was placed in the superficial femoral artery, ideally at the time of VA-ECMO institution. Whenever feasible, image-guided cannulation using vascular ultrasound was employed during percutaneous access. The ECMO circuits in use were biocompatible heparin-coated, comprising of two cannulas, an integrated centrifugal pump and a polymethylpentene membrane oxygenator, with 3/8″ connecting tubes (HLS Set Advanced 7.0^®^; Maquet-Cardiopulmonary; Hirrlingen, Germany).

Bilateral lower limb tissue saturation was monitored using Near-Infrared Spectroscopy during VA-ECMO support, extending to at least 24 h post-decannulation. A protocolized anticoagulation therapy with unfractionated heparin to an activated partial thromboplastin time of 1.5 normal was routinely used during VA-ECMO. Daily dressing changes of the arterial cannula were performed, along with daily transthoracic or transoesophageal echocardiography including assessment of distal perfusion cannula patency and flow characteristics.

### General management protocol

Patients eligible for liberation from VA-ECMO were divided into PP post-closure group and compared with a partially historical cohort of conventional care, which included surgical arteriotomy repair and manual compression. No clinical characteristics, besides femoral artery extreme calcification, were considered strict contraindications to percutaneous closure. Vascular Surgeons and Intensive Care physicians shared the decision of whether to perform open surgery or a percutaneous closure, at physicians’ discretion, considering individual experience and the patient’s haemorrhagic risk. When there was heightened concern for significant complications or atherosclerotic disease, contralateral vessels were evaluated pre-procedure using ultrasound or, if clinically feasible, computer tomography. This assessment aimed to estimate the ipsilateral atherosclerotic burden. Common femoral artery (CFA) diameter measurement is not mandatory, and was not part of the study’s protocol, as the patient’s pool included emergent situations where time is critical, and the experienced ECMO teams often rely on clinical assessment to choose appropriate cannula sizes without formal CFA measurement. Although not mandatory, CFA diameter measurement is highly advisable, especially in elective ECMO cases, to optimize cannulation and reduce risks. Patients with thrombocytopenia or severe coagulation alterations were optimized as possible.

Bed-side percutaneous femoral artery closure was performed using a PP post-closure technique, with the procedure performed in collaboration with a Vascular Surgeon, having two units of red blood cells on standby. Three operators are required, one for the percutaneous closure, another guiding the bedside ultrasound and a third controlling the bleeding. After a sterile area preparation, both arterial and venous cannulas were clamped, the arterial cannula punctured proximally, a standard 150 cm guidewire was inserted, and the arterial cannula was removed along the guidewire. Usually, two PP devices were inserted over the guidewire deployed at 10 and 2 o’clock. A third one was placed if haemostasis was inadequate. Following the procedure Doppler ultrasound was routinely performed at 0 and 24 h to rule out complications.

In the conventional approach (control group), VA-ECMO was stopped, both cannulas removed, and manual compression applied until haemostasis.

### Statistical analysis

For statistical analysis, SPSS (IBM Corp., released 2022. IBM SPSS Statistics for Windows, version 29.0, Armonk, NY, USA) was used. Data was presented as *n* (%) or reported using mean and standard deviation or median and interquartile range, depending on normality assumptions. Student’s *t*-test was favoured when dealing with normally distributed continuous variables. At the same time, the Mann-Whitney-*U* was privileged when working with variables whose normal distribution could not be assumed. The Chi-squared test was used to analyse categorical variables. Statistically significant results were set at a *p* < 0.05 level.

## Results

**Figure 2.**
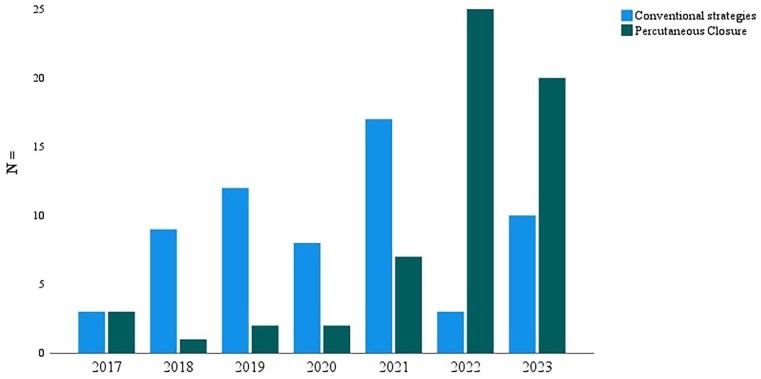
VA-ECMO decannulation strategies by year. PP: Perclose Proglide post-closure; VA-ECMO: venoarterial extracorporeal membrane oxygenation.

A total of 201 patients underwent VA-ECMO during the study period, with 122 (61%) meeting the inclusion criteria. Among them, 60 (49.2%) patients comprised the PP group and 62 (50.8%) underwent conventional decannulation, 50 managed with manual compression and 12 with open surgical repair (Figure 1). The PP post-closure technique has gained momentum over the years, with the trends in decannulation strategies depicted in Figure 2.

The demographic characteristics of the patients are shown in Table 1. The PP group consisted of older individuals (51.4 vs 45.9 years of age, *p* = 0.021), with a higher prevalence of arterial hypertension compared to the control group (46.7% vs 29%, *p* = 0.045). Only 5.7% of patients had a history of peripheral arterial disease (PAD), but most patients presented at least one cardiovascular risk factor (97.5%). There were no differences in the remaining baseline characteristics. Cardiogenic shock following acute myocardial infarction (AMI-CS) and extracorporeal cardiopulmonary resuscitation (ECPR) were the primary diagnosis for the introduction of VA-ECMO. There were no significant differences in the initial physiological insult severity between groups. The majority of VA-ECMO cannulations occurred in the ICU setting (42.6%), followed by the emergency room (30%). The most frequent arterial cannulas used were 15–17 Fr in size. Patients in the PP were more likely to have smaller size arterial cannulas (14.86 ± 0.977 Fr vs 15.56 ± 0.917 Fr, *p* = 0.009) and to have had their cannulation done with image-guided vascular ultrasound (58.3% vs 38.7%, *p* = 0.030). One patient had a 19 Fr cannula inserted and was decannulated with PP. Four patients had 13 Fr size cannulas inserted, and were all decannulated with PP.

**Table 1.** Baseline characteristics, primary diagnosis, disease severity and cannulation features.

Population chacteristics	Control group	PP group	*p*
	*n* = 62 (50.8%)	*n* = 60 (49.2%)	
Age, years (mean ± SD)	45.9 (±13.6)	51.4 (±11.9)	0.021
Male, *n* (%)	42 (67.7%)	36 (60%)	0.373
Cardiovascular risk factors			
Smoking	24 (38.7%)	21 (35%)	0.671
Dyslipidaemia	19 (30.6%)	24 (40%)	0.280
Hypertension	18 (29%)	28 (46.7%)	0.045
Diabetes mellitus	7 (11.3%)	12 (20%)	0.185
Obesity (BMI >30 kg/m^2^)	10 (16.1%)	9 (15%)	0.863
Peripheral artery disease	3 (4.8%)	4 (6.7%)	0.664
Chronic kidney disease	1 (1.6%)	0 (0)	0.238
Primary diagnosis/VA-ECMO indication			
AMI-CS	16 (25.8%)	10 (16.7%)	0.347
ECPR	11 (17.7%)	10 (16.7%)	
Arrhythmic storm	9 (14.5%)	4 (6.7%)	
Myocarditis	8 (13.0%)	9 (15%)	
Postcardiotomy cardiogenic shock	5 (8.1%)	6 (10%)	
Pulmonary embolism	5 (8.1%)	3 (5%)	
Terminal heart failure	2 (3.2%)	4 (6.7%)	
Other	6 (9.7%)	14 (23%)	
Disease severity			
APACHE II (mean ± SD)	22.7 (±7.251)	22.3 (±8.460)	0.769
SAPS II (mean ± SD)	51.6 (±15.43)	49.3 (±16.901)	0.448
Lactic acid, mmol/L (mean ± SD)	6.2 (±4.4)	5.56 (±3.8)	0.372
Setting of VA-ECMO cannulation			0.151
ICU	28 (45.2%)	24 (40%)	
Emergency room	14 (22.6%)	23 (38.3%)	
Operation room/Cath lab	9 (14.5%)	6 (10%)	
Wards	2 (3.2%)	4 (6.7%)	
Other hospitals	9 (14.5%)	3 (5%)	
Arterial cannula size, Fr (mean ± SD)	15.56 (±0.917)	14.86 (±0.977)	0.009
Ultrasound guided cannulation	24 (38.7)	35 (58.3)	0.030

AMI-CS: cardiogenic shock after acute myocardial infarction; BMI: body mass index; ECPR: extracorporeal cardiopulmonary resuscitation; Fr: French; SD: standard deviation; PP: Perclose Proglide^®^ suture-mediated vascular closure device; VA-ECMO: venoarterial extracorporeal membrane oxygenation.

The median time spent on VA-ECMO for the PP group was 8 days [interquartile range 5–12], a shorter period than the control group 13.5 [6–24.5]. In both groups liberation from VA-ECMO occurred after cardiac function recovery in most patients (81%). The PP group had a lower mean platelet count (120.9 × 10^9^/µL vs 159 × 10^9^/µL, *p* = 0.024), but both groups’ haemoglobin levels and coagulation studies were similar. Most patients received antithrombotic or anticoagulation therapy at decannulation from VA-ECMO. There were no differences between the two groups regarding antiplatelet or anticoagulation use or infection burden at the time of decannulation (Table 2).

**Table 2.** Patients’ characteristics at the timing of decannulation from VA-ECMO.

Decannulation conditions	Control group	PP group	*p*
	*n* = 62 (50.8%)	*n* = 60 (49.2%)	
VA-ECMO duration, days [median + IQR]	13.5 [6–24.5]	8 [5–12]	0.021
Indication for VA-ECMO discontinuation			0.679
Recovery	50 (60.8%)	50 (83.3%)	
Heart transplant	11 (11.7%)	8 (13.3%)	
Other	1 (1.6%)	2 (3.3%)	
Anticoagulation	47 (75.8%)	41 (68.3%)	0.351
Platelet antiaggregation	20 (32.3%)	16 (26.7%)	0.498
Active infection	32 (51.6%)	28 (46.7%)	0.585
Haemoglobin, g/dL (mean ± SD)	9.7 (±5.4)	8.15 (±0.9)	0.407
Platelets count, 10^9^/µL (mean ± SD)	159 (±118.0)	120.9 (±91.4)	0.024
APTT, s (mean ± SD)	40.83 ± 9.7	40.46 ± 10.685	0.844
Reperfusion cannula size [IQR]	7 [6.25-7]	7 [6.25-7]	

APTT: activated partial thromboplastin time; IQR: interquartile range; SD: standard deviation; VA-ECMO: venoarterial extracorporeal membrane oxygenation.

The number of patients free from decannulation-related adverse events was larger in the PP cohort (66.9% vs 54.9%). Decannulation related vascular complications were less frequently reported in the percutaneous closure group, who also had higher haemoglobin level 24 h post decannulation (8.6 ± 0.8 vs 8.3 ± 0.9, *p* = 0.047), with no difference in terms of transfusion of red blood cells between groups. The pseudoaneurysm was the most frequently reported complication in both groups, followed by limb ischaemia. Infection of the arterial cannulation site was documented in two patients, both of whom underwent surgical repair (Table 3).

**Table 3.** Clinical outcomes comparison per arterial closure strategy.

Postoperative Outcomes	Control group	PP group	*p*	*P*
	*n* = 62 (50.8%)	*n* = 60 (49.2%)		
Decannulation related adverse events				
None	34 (54.9%)	40 (66.7%)	0.181	0.091
Pseudoaneurysm	14 (22.6%)	9 (15%)	0.285	
Limb ischaemia	7 (11.3%)	5 (8.3%)	0.583	
Arterial thrombosis	4 (6.5%)	3 (5%)	0.730	
Haemorrhage	0 (0)	2 (3.3%)	0.147	
Haematoma	0 (0)	1 (1.7%)	0.307	
Fistula	3 (2.5%)	0 (0)	0.084	
Surgical site infection	2 (3)	0	0.161	
Post-decannulation				
Haemoglobin 24 h after decannulation (mean ± SD)	8.3 (±0.9)	8.6 (±0.8)	0.047	
Δ Haemoglobin (mean ± SD)	−0.4548 (±5.7)	+0.4450 (±0.8)	0.230	
Red blood cell transfusion (mean ± SD)	0.7 (±0.8)	0.53 (±0.8)	0.263	
ICU days post-decannulation (median + IQR)	11 (5–25)	8 (4–18.75)	0.312	
Inpatient ward days post-decannulation (median + IQR)	26 (13.5–50)	19 (8–54)	0.307	
30 days mortality	4 (6.6%)	7 (11.9%)	0.314	

Δ Haemoglobin: pre-post decannulation haemoglobin; IQR: interquartile range; SD: standard deviation; ICU: intensive care unit instay; PP: percutaneous; VA-ECMO: venoarterial extracorporeal membrane of oxygen.

A trend towards reduced ICU and hospital length of stay after decannulation in the PP group was noted, although it did not reach statistical significance. Seven patients (11.9%) died in the PP cohort and 4 (6.6%) in the control group, and there were no significant differences between both groups (*p* = 0.314). No procedure-related deaths occurred.

The overall safety and performance of the procedure was measured by device failure. A mean of 2.7 (±1) PP devices were required to achieve complete haemostasis in the PP cohort, where technical failure was documented in four cases (6.7%). One patient was managed conservatively with conventional cerclage and manual compression, and three patients required immediate conversion to surgical decannulation and repair.

In the PP cohort, twenty patients developed decannulation-related adverse events (Table 3), eight patients required surgical repair: six for acute limb ischaemia immediately after decannulation and two for pseudoaneurysms about 3 days later. In the control group, 12 patients with severe thrombocytopenia (platelet counts <10,000 × 10^9^/µL) were decannulated using surgical arteriotomy repair, in the operating theatre without associated complications. It was notable that 16 patients in manual compression subgroup experienced vascular complications requiring surgical repair.

## Discussion

Percutaneous vascular closure devices offer a minimally invasive alternative to manual compression or open surgical repair for arterial closure at VA-ECMO decannulation. The study demonstrates the effectiveness and safety of PP vascular devices with a post-closure technique for VA-ECMO decannulation. The results reveal a low rate of device failure and vascular decannulation-related complications. Compared with conventional strategies, in the PP group a trend towards reduced ICU and hospital length of stay after decannulation was noted, although it did not reach statistical significance. No procedure-related deaths occurred. There were no significant differences in all-cause mortality 30 days after ICU discharge.

Despite being widely used in various clinical settings,^6,7,9^ the use of PP for VA-ECMO decannulation has only been reported in small case series or observational cohorts, with an overall high risk of bias. Moreover, clinical outcomes remain poorly documented, as most studies have focused on procedural aspects, such as time, showing shorter procedure time with percutaneous artery closure.^10^

The rates of device failure (6.7%), closely match those found in other research studies for different endovascular procedures, including endovascular aortic repair and transcatheter aortic valve replacement.^11–13^ The PP features a significant learning curve, emphasizing the importance of physician’s skill and experience for technical success. Moreover, device failure rates have shown a decreasing trend over time as operator experience evolves.^11–13^ The authors propose that the study period coincident with the centre’s learning may underestimate the technique’s full potential impact, due to inherent complications associated with the learning phase.

Managing arterial access after VA-ECMO poses greater complexity, owing to prolonged duration of support, ECMO-associated coagulopathy,^14^ and risk of arterial thrombosis in the low-flow space between arterial and distal reperfusion cannulas.^15^ While some studies have employed a pre-closure technique,^4^ where the suture is placed around the arteriotomy before cannulation, this may delay VA-ECMO initiation and increase the risk of subsequent wound infection and closure failure.^16^ In this setting the author’s experience favours a post-closure technique with similar efficacy, in line with the available literature, which reports similar effectiveness for both pre and post-closure techniques in VA-ECMO decannulation^16–18^ and other clinical scenarios.^11–13^

PP percutaneous artery closure appears efficacious for patients with shorter runs of ECMO support and smaller cannulas (15–17 Fr), often inserted with ultrasound guidance. The improved cannulation conditions, enhance the success of a minimally invasive decannulation. This aligns with existing literature reporting that artery diameter and difficult cannulations, with risk of vessel injury, are important risk factors for vascular complications.^15^ Surgical closure offers the advantage of closing the access under direct visualization and holds a role for a restricted pool of patients with poor anatomical conditions.^14^

Decannulation-related adverse events were reduced in the PP group, aligning with findings in other clinical scenarios, where PP have high technical success rate with significantly lower overall adverse events.^6,7,9^ Pseudoaneurysm emerged as the most documented decannulation-related vascular complication. Particularly in cases of prolonged VA-ECMO support, where there’s an increased risk of incomplete arteriotomy sealing by PP, pseudoaneurysms are a concern. Even though, pseudoaneurysms were less frequently reported in the PP group,^14^ which could suggest a potential protective effect.

While low PAD prevalence may explain low vascular complication rates, most patients presented at least one cardiovascular risk factor. Older age and hypertension are consistently associated with increased vascular complications during VA-ECMO decannulation.^15^ However, the PP cohort presented with lower decannulation-related adverse events, despite the higher prevalence of older individuals with arterial hypertension.

Only two access site infections were reported, both in the open surgical repair subgroup. This is consistent with existing literature suggesting a link between vascular closure devices and decreased infection rates, likely attributed to the minimal incision, enhanced patient comfort and early ambulation.^6^ Surgical closure, on the other hand, may delay wound healing and increase infection risk.^16^

Although not warranting an active alternative therapeutic approach, the PP cohort presented mild thrombocytopenia. Despite this, there were no differences in terms of bleeding events, haemoglobin levels 24 h post-decannulation or red blood cell transfusion requirements between both groups. These findings are consistent with those of smaller patient’s series, demonstrating reduced bleeding events and packed red blood cell usage in VA-ECMO patients decannulated with PP.^4^ Notably, anticoagulation and platelet antiaggregation did not differ between the groups. Anticoagulation is used to reduce circuit-associated thrombotic risk,^19^ however this uniform practice might potentially contribute to increased rates of haemorrhagic complications.

Although not reaching statistical significance, a trend towards shorter ICU and hospital length of stay are anticipated with percutaneous decannulation. The reduce incidence of short-term complications strongly suggest that using PP could reduce length hospital and ICU stay. Literature comparing economic outcomes of PP with conventional approaches also supports this notion, showing significantly lower hospitalization costs with PP percutaneous decannulation.^20^ Considering that ICU costs are the greatest in the patients’ course of hospital admission, PP percutaneous decannulation emerges as a potential cost-saving strategy.

No procedure-related deaths occurred and there were no significant differences in all-cause mortality 30 days after ICU discharge. The existing literature is scarce but considering the absence of reported procedure-related deaths, this might constitute another indicator attesting to the safety of PP artery closure during VA-ECMO decannulation.^16,21,22^

This study has several limitations. Firstly, is a single-centre retrospective study, which makes it open to bias, resorting to a partially historical cohort for control. The cumulative experience of the group might have independently mitigated adverse events. Further synthesis studies may be sufficient to validate these findings and large prospective multicentre randomized trial might be unnecessary. Secondly, the study focuses on immediate outcomes and compares percutaneous closure to conventional approaches, including manual compression and surgical repair, representing distinct strategies. The study paralleled the learning curve of intensivist doctors, with increased risk of adverse events despite the supervision of vascular surgeons. Procedural proficiency underscores the crucial role of physician expertise for achieving technical success. While the authors use PP vascular devices, the optimal choice among the available devices requires further investigation. Lastly, long-term outcomes associated with the use of PP post-closure technique in VA-ECMO decannulation remain unclear, although other areas, such as Transcatheter Aortic Valve Implantation and Endovascular Aortic Repair failed to report adverse events.^23,24^

Despite the limitations, the study comprises an extensive series of consecutive patients decannulated from VA-ECMO with percutaneous femoral artery arterial closure. Its uniqueness lies in the comprehensive assessment of vascular complications associated with VA-ECMO decannulation. Bedside percutaneous artery closure for VA-ECMO decannulation is safe and effective. It holds the potential to decrease cost-burden for both patients and hospitals, while reducing the strain on operating theatres and surgical teams, lessen manpower requirements, sparing patients from high-risk transport. However, these benefits were not tangible on this work.

## Conclusion

Suture-mediated closure devices are safe and effective in femoral artery percutaneous closure during VA-ECMO decannulation, with few decannulation-related adverse events and a low device failure rate. Bedside decannulation has the potential to reduce the cost burden for both patients and hospitals while maintaining safe outcomes, as many of the benefits from this technique still need to be fully captured by the measured outcomes. Synthesis studies are required in order to validate these findings and facilitate their incorporation into clinical practice and guidelines.
